# Saikosaponin A, a Triterpene Saponin, Suppresses Angiogenesis and Tumor Growth by Blocking VEGFR2-Mediated Signaling Pathway

**DOI:** 10.3389/fphar.2021.713200

**Published:** 2021-10-29

**Authors:** Pan Zhang, Xing Lai, Mao-Hua Zhu, Mei Long, Xue-Liang Liu, Zi-Xiang Wang, Yifan Zhang, Run-Jie Guo, Jing Dong, Qin Lu, Peng Sun, Chao Fang, Mei Zhao

**Affiliations:** ^1^ Department of Pharmacy, Shanghai University of Medicine and Health Sciences, Shanghai, China; ^2^ Graduate School, Shanghai University of Traditional Chinese Medicine, Shanghai, China; ^3^ Tongren Hospital and State Key Laboratory of Oncogenes and Related Genes, Department of Pharmacology and Chemical Biology, Hongqiao International Institute of Medicine, Shanghai Jiao Tong University School of Medicine (SJTU-SM), Shanghai, China; ^4^ Department of General Surgery, Tongren Hospital, SJTU-SM, Shanghai, China

**Keywords:** saikosaponin A, angiogenesis, VEGFR2, chick embryo chorioallantoic membrane, cancer therapy

## Abstract

Saikosaponin A (SSA), a main triterpenoid saponin component from *Radix Bupleurum*, has been revealed to have a variety of pharmacological activities. However, whether SSA can inhibit angiogenesis, a key step in solid tumor progression, remains unknown. In this study, we demonstrated that SSA could powerfully suppress the proliferation, migration, and tube formation of human umbilical vein endothelial cells. SSA also significantly inhibited angiogenesis in the models of the chick embryo chorioallantoic membrane and Matrigel plugs. Moreover, SSA was found to inhibit tumor growth in both orthotopic 4T1 breast cancer and subcutaneous HCT-15 colorectal tumor by the inhibition of tumor angiogenesis. Western blot assay indicated the antiangiogenic mechanism of SSA in the suppression of the protein phosphorylation of VEGFR2 and the downstream protein kinase including PLCγ1, FAK, Src, and Akt. In summary, SSA can suppress angiogenesis and tumor growth by blocking the VEGFR2-mediated signaling pathway.

## Introduction

**FIGURE 1:**
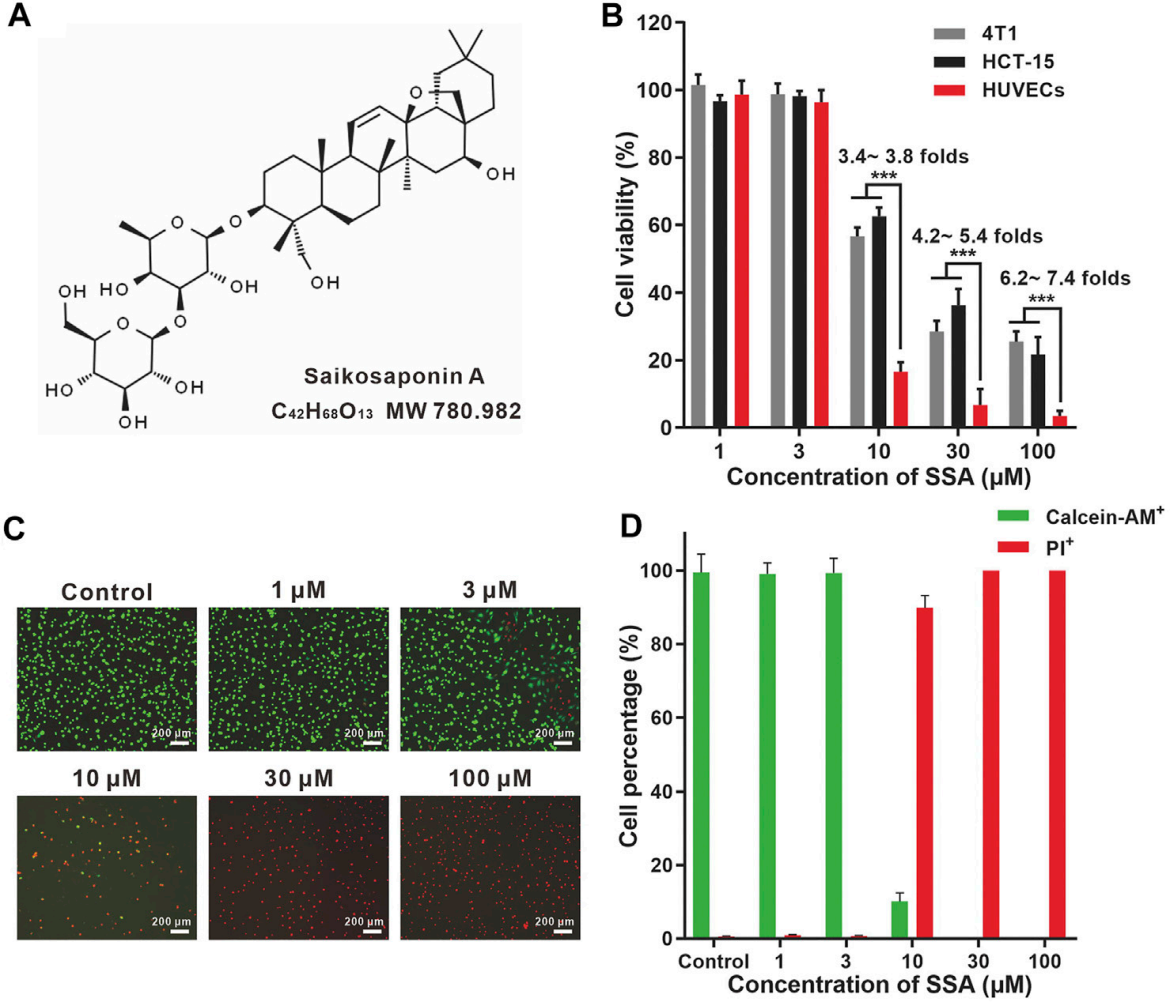
SSA more efficiently suppressed the viabilities of HUVECs compared to those of HCT-15 and 4T1 cells. **(A)** Chemical structures of SSA. **(B)** Effect of SSA on cell viability. **(C)** Representative fluorescence photographs of HUVECs stained with calcein-AM/PI after treatment with SSA. **(D)** The live (calcein^+^) and dead (PI^+^) cell percentages of HUVECs after SSA treatments. All values were expressed as mean ± *s*.d. *n* = 4. ***p < 0.001.

Saikosaponin A (SSA, Figure 1A), a main triterpenoid saponin component from *Radix Bupleurum*, has been widely investigated for its multiple pharmacological activities, such as antidepressant (Guo et al., 2020), immunoregulatory (Qi et al., 2021), and anti-inflammatory properties (He et al., 2016; Du et al., 2018; Zhou et al., 2019; Piao et al., 2020). The anticancer effects of SSA are also intriguing with diverse molecular mechanisms. Specifically, SSA can induce apoptosis or inhibit the proliferation of tumor cells through caspase-2, -4, and -8 activation (Kim and Hong, 2011; Kang et al., 2017); ERK signaling activation; or CXCR4 downregulation (Wang et al., 2019). SSA also regulates Th1/Th2 balance in tumors (Zhao et al., 2019) and generates microbicidal neutrophils to reduce cancer chemotherapy–induced neutropenia infection (Qi et al., 2021); both of them can benefit from antitumor therapy. However, it remains elusive whether SSA can directly suppress angiogenesis, the hallmark and a key step in solid tumor progression (Hanahan and Weinberg, 2011).

Angiogenesis, the development of new blood vessels from pre-existing ones, is a validated target in cancer clinics. More than 10 antiangiogenic drugs, including small kinase inhibitors (sunitinib, sorafenib, pazopanib, etc.), antibodies (bevacizumab and ramucirumab), and fusion proteins (aflibercept), have been approved by the FDA and other countries for multiple cancer indicators (Jayson et al., 2016). However, low efficacy, toxic side effects, unsatisfied pharmacokinetic behavior, and high cost limit their wide use in cancer clinics (Jain et al., 2011; Jayson et al., 2016; Kim et al., 2019). It is urgent to develop new antiangiogenic agents. In recent years, exploring antiangiogenic natural products is emerging as an attractive research field. Many natural products with diverse molecular structures exerted potent antitumor actions via multiple antiangiogenic mechanisms (Guan et al., 2016).

In this work, the antiangiogenic effect of SSA and its molecular mechanism were revealed. SSA suppressed the proliferation, migration, and tube formation of human umbilical vascular endothelial cells (HUVECs, a classical *in vitro* cell model mimicking tumor vascular endothelial cells). SSA inhibited angiogenesis in the chick embryo chorioallantoic membrane (CAM) and Matrigel plug models. Moreover, SSA suppressed angiogenesis and tumor growth in orthotopic 4T1 breast cancer and subcutaneous HCT-15 xenograft in mice without overt toxicity. The underlying molecular mechanism of SSA is VEGFR2 signal blocking, which was proved in Western blot assay.

## Materials and Methods

### Materials, Cells, and Animals

Saikosaponin A (SSA) was purchased from Push Bio-Technology Company (Chengdu, China). Dulbecco’s modified Eagle medium (DMEM), RPMI 1640 medium, fetal bovine serum (FBS), penicillin, and streptomycin were provided by Basal Media Technologies (Shanghai, China). The recombinant human vascular endothelial growth factor (VEGF_165_) was obtained from ProSpec-Tany TechnoGene (Ness Ziona, Israel). The Matrigel matrix was obtained from BD Biosciences (San Jose, CA, United States). Antibodies for Western blotting in the VEGFR2 signaling assay were supplied by Cell Signaling Technology (Shanghai, China). The antibodies were VEGF receptor 2 (55B11) rabbit mAb (#2479), phospho-VEGF receptor 2 (Tyr1175) rabbit mAb (#2478), PLCγ1 rabbit antibody (#2822), phospho-PLCγ1 (Ser1248) (D25A9) rabbit mAb (#8713), FAK rabbit antibody (#3285), phospho-FAK (Tyr397) (D20B1) rabbit mAb (#8556), Src rabbit antibody (#2108), phospho-Src Family (Tyr416) (E6G4R) rabbit mAb (#59548), Akt (pan) (11E7) rabbit mAb (#4685), and phospho-Akt (Ser473) (D9E) XP rabbit mAb (#4060). Goat anti-rabbit IgG H&L (HRP) (ab205718) was supplied by Abcam (Shanghai, China).

Primary human umbilical vascular endothelial cells (HUVECs) were obtained from Lifeline Cell Technology (Frederick, MD). HUVECs were cultured in the VascuLife VEGF Cell Culture Medium (Frederick, MD), which contained supplements and growth factor cytokines, including VEGF, EGF, IGF-1, and bFGF. The HCT-15 human colorectal adenocarcinoma cell line and 4T1 mouse breast cancer cell line were purchased from American Type Culture Collection (Manassas, VA). They were all cultured at 37°C in humidified atmosphere containing 5% CO_2_.

BALB/c mice or nude mice (20 ± 2 g) were provided by Shanghai Laboratory Animal Center (Chinese Academy of Sciences, Shanghai, China). All animal-associated experiments in this study were approved by the Ethical Committee of Shanghai Jiao Tong University School of Medicine.

### Cell Viability Assay

The effect of SSA on cell viability was analyzed by using the Cell Counting Kit-8 (Dojindo Laboratories, Kumamoto, Japan). HUVECs, 4T1 or HCT-15 cells, were seeded at a density of 6 × 10^3^ cells/well in 96-well plates (Corning, United States). After 24-h incubation, the cells were treated with various concentrations of SSA (1–100 μM) for 48 h. Then 10 μL CCK-8 solution was added for an additional 2-h incubation at 37°C. All experiments were performed in triplicate. Absorbance at 450 nm was measured using a microplate reader. Cell viability (%) was calculated against the control.

The LIVE/DEAD cell viability/cytotoxicity kit (Life technology, Carlsbad, CA) was also used to assess the viability of HUVECs. Calcein-AM can be transformed into calcein with green fluorescence in live cells, and propidium iodide (PI, red fluorescence) can stain the nuclei of dead cells. HUVECs (6 × 10^3^ cells/well) were cultured in 96-well plates. After incubation at 37°C for 12 h, the medium was replaced by 200 μL of 1–100-μM SSA for 48-h incubation. Then the culture medium was replaced with 1 ml PBS containing 2 μM calcein-AM (Ex 488 nm and Em 515 nm) and 4.5μM PI (Ex 535 nm, Em 615 nm) for 15 min to stain live and dead cells.

### Wound Healing Assay

HUVECs were seeded in 96-well plates (1 × 10^4^ cells/well) and allowed to grow to confluence. Then the cells were scratched with WoundMaker (IncuCyte) and treated with various concentrations of SSA (1–100 μM) for 12 h. Then the cells were incubated with fresh medium till 48 h. After 48 h, the cells were observed and photographed using the IncuCyte Live-Cell Analysis System (Essen BioScience). Cell migration was quantified using Image-Pro Plus 8.0 software (Media Cybernetics, Bethesda, MD).

### Transwell Migration Assay

The HUVEC migration assay was carried out in a 96-well Transwell Boyden chambers with a polycarbonate filter of a pore size of 8 μm and 6.5 mm diameter inserts (Corning Costar, MA). In brief, 5 × 10^5^ cells suspended in 100-μL serum-free medium with various concentrations of SSA (1–100 μM) were added to the upper chamber. The bottom chambers were filled with 600 μL completed endothelial cell medium containing 20 ng/ml VEGF_165_. The cells were cultured routinely in an incubator (37°C with 5% CO_2_). After 10 h, the upper surface of the membrane was gently wiped with a cotton swab to remove non-migrating cells. The membrane was then fixed in 4% glutaraldehyde for 20 min and stained with crystal violet overnight at room temperature. After washing the Transwell chamber five times with PBS, the membrane was photographed using an EVOS microscope (Life Technologies, Grand Island, NY). The migrated cells were quantified using Image-Pro Plus 8.0 software.

### Endothelial Cell Tube Formation Assay

Chilled Matrigel (BD Biosciences, CA) was thawed overnight at 4°C, dispensed into 96-well plates (70 μL/well), and then incubated at 37°C for 30 min for solidification. Then approximately 2 × 10^5^ cells suspended in 100 μL medium containing various concentrations of SSA (1–100 μM) were seeded on Matrigel. After 8 h, the images of HUVEC tubular structures were photographed using the EVOS microscope, and the tube length and inhibition effect were analyzed using Image-Pro Plus 8.0 software.

### Chick Embryo Chorioallantoic Membrane Assay

To evaluate the *in vivo* angiogenic effect of SSA, the chick embryo CAM assay was applied in this experiment (Liu et al., 2018). In brief, the fertilized eggs were placed in an incubator with approximately 60% humidity and 37.8°C. After 8 days, ∼ a 1-cm^2^ window was opened, and the shell membrane was removed to expose the CAM. A sterilized 5-mm diameter Whatman filter sheet as a drug carrier that was soaked with SSA of concentrations (1–100 μM) was placed on the CAM. The saline group was included as the control. Then the window was sealed with parafilm and returned to the incubator for additional 48 h. The images of CAM were captured through the windows using a digital camera (Nikon, Japan), and the neovascularization was quantified using Image-Pro Plus 8.0 software.

### Matrigel Plug Assay

The *in vivo* antiangiogenic activity of SSA was also evaluated in the Matrigel plug model (Liu et al., 2015). Female BALB/c mice were divided into five groups (*n* = 4) and subcutaneously injected with 500-μL Matrigel that contained 30 U heparin with recombinant human VEGF_165_ (50 ng/ml) and various concentrations of SSA (0, 3, 10, 30 μM). Matrigel containing no VEGF_165_ (50 ng/ml) was set as control. After 12 days, the mice were euthanized. The Matrigel plugs were harvested, fixed in 4% glutaraldehyde overnight, and processed for CD31 immunofluorescence staining. The images of microvessels were photographed under the EVOS microscope, and microvessel density (MVD) was quantified using Image-Pro Plus 8.0 software.

### Anticancer Therapy of SSA in Orthotopic 4T1 Tumor in Mice

One million 4T1 cells were injected into the right mammary fat pad of female BALB/c mice to establish the orthotopic tumor model. When the tumor grew to approximately 100 mm^3^, 16 mice were divided into two groups and treated intraperitoneally with saline or SSA (10 mg/kg/day) for consecutive 15 days. The tumor volume and body weight were recorded every day. The tumor volume was calculated as follows: volume = (length×width^2^)/2. On the final day, the mice were euthanized, and the tumors were removed and weighted. The tumors were fixed in 4% paraformaldehyde solution and processed for frozen sections. Then the slices were stained with rat anti-mouse CD31 antibody (1: 200, BD Biosciences, Shanghai, China) and Cy3 conjugated goat anti-rat IgG (H + L) (1:300, Servicebio, Wuhan, China) (Ex 550 nm, Em 570 nm) for microvessel density (MVD) assay. The tumor tissues were also processed for paraffin sections for pathological examination. The sections were then stained with anti-Ki-67 rabbit pAb (1:500, Servicebio, Wuhan, China) and HRP-conjugated goat anti-rabbit IgG (H + L) (1:200, Servicebio, Wuhan, China). Then the slices were photographed under a confocal laser scanning microscope (Leica TCS-SP8) or photomicroscope (Leica DFC 320), and the images were analyzed for microvessel density (MVD), Ki-67–positive tumor cells, and tumor necrosis area using Image-Pro Plus 8.0 software.

### Anticancer Therapy of SSA in Subcutaneous HCT-15 Tumor in Nude Mice

Three million HCT-15 cells were subcutaneously injected into the right anterior axillary of the six-week-old BALB/c nude mice to establish the xenograft tumor model. When the tumor volume reached ∼100 mm^3^, 16 mice were randomly divided into two groups. Then the mice were treated intraperitoneally with saline or SSA (10 mg/kg) every day consecutively for 15 days. The methods for antitumor evaluation are same as those in the aforementioned anti-4T1 tumor experiment.

### Western Blot

Western blot assay was used to investigate the antiangiogenic mechanism of SSA. In brief, HUVECs (2 × 10^5^ cells per well) were seeded in 6-well plates and incubated for 2 days until they reached 80% confluence. Then the cells were incubated with various concentrations of SSA for 30 min and stimulated with 50 ng/ml VEGF_165_ for 4 min. Subsequently, RIPA lysis buffer (Beyotime, Shanghai, China) supplemented with PMSF (Sangon Biotech, Shanghai, China) and phosphatase inhibitor cocktail (EpiZyme, Shanghai, China) were added to each well to extract whole cell lysates. The BCA Protein Quantification Kit (Yeasen biotech, Shanghai, China) was used to determine the protein concentration. Equal amounts of protein (30 μg) were applied to 10% SDS-PAGE, and they were transferred onto a PVDF membrane (Millipore, Bill-erica, MA). The membrane was then blocked in 5% non-fat milk blocking buffer for 1 h and incubated with specific primary antibodies (1:1,000, Cell Signaling Technology, Shanghai, China) followed by exposure to HRP-conjugated secondary antibody (1:5,000, Abcam, Shanghai). All experiments were carried out at least three times.

### Statistical Analysis

All data obtained were presented as mean ± *s*.d. Statistical analysis was performed using GraphPad Prism 8.0 (GraphPad Software, San Diego, CA). Differences between the groups were examined using one-way ANOVA with Bonferroni’s multiple comparison tests or Student’s t test. The differences were considered significant when the *p* value was below 0.05.

## Results

### SSA Inhibited the Viability of Human Umbilical Vascular Endothelial Cells More Efficiently Than That of 4T1 and HCT-15

HUVEC is a normal cell line that can mimic tumor endothelial cells well. (Guan et al., 2015). SSA efficiently suppressed HUVEC viability in a dose-dependent manner (Figure 1B). The viabilities of HUVECs were completely suppressed when SSA concentrations exceeded 30 μM. Similar observations were obtained in the calcein-AM and PI dual staining assay (Figures 1C,D). Compared to HUVECs, tumor cells (4T1 and HCT-15) were less sensitive to SSA. 3.4- ∼ 7.4-fold higher viability of the 4T1 and HCT-15 cells was maintained after treatment with 10–100 μM SSA. Generally, the activated tumor endothelial cells are more sensitive to chemotherapeutic drugs than tumor cells, offering a choice for specific antiangiogenic therapy (Kerbel and Kamen, 2004). We also examined the cytotoxicity of SSA in two normal cell lines, L-02 (human fetal hepatocyte line) and ARPE-19 (a human retinal pigment epithelial cell line). It showed that compared to HUVECs, L-02 and ARPE-19 were less sensitive to SSA (Supplementary Figure S1), suggesting the potential safety of SSA when used *in vivo*.

### SSA Inhibited Human Umbilical Vascular Endothelial Cell Migration and Tube Formation

**FIGURE 2:**
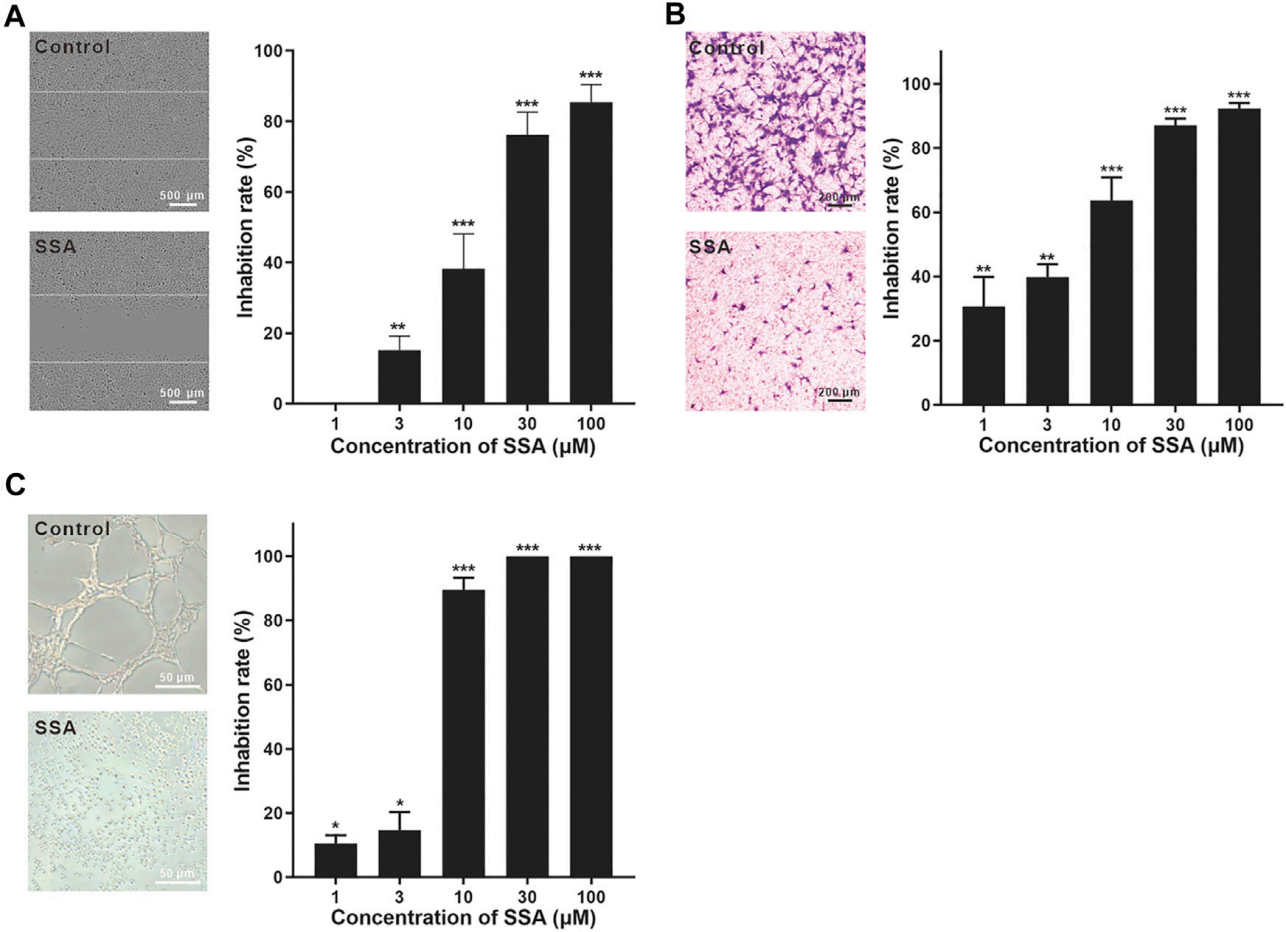
SSA significantly suppressed HUVEC migration and tube formation. **(A)** SSA suppressed the horizontal migration of HUVECs in the wound healing test. The line indicated the initial boundary of the cells. **(B)** SSA inhibited HUVEC migration in the Transwell assay. The migrated cells were stained with crystal violet and quantified using Image-Pro plus 8.0 software. **(C)** SSA inhibited HUVEC tube formation. The length of the tubular structures was photographed and quantified using Image-Pro plus 8.0 software. The photos of the control and SSA groups (30 μM) were shown in each panel. The values are expressed as mean ± *s*.d. *n* = 3. *p < 0.05, **p < 0.01, and ***p < 0.001. SSA suppressed angiogenesis in the chick embryo CAM.

The wound healing test is a representative method for the evaluation of HUVEC migration (Guan et al., 2015; Li et al., 2019). HUVECs were pretreated with SSA for 12 h and then incubated in fresh medium till 48 h. After 12-h incubation, more than 65% cells were still alive even at the highest test concentration (100 μM) (Supplementary Figure S2). The inhibition of the horizontal migration of HUVECs in the wound healing test was obtained in a dose-dependent manner. ∼40% migration inhibition was obtained under 10 μΜ SSA, and this effect increased to nearly 80% when 30 μΜ or higher (100 μΜ) SSA was used (Figure 2A). It is noted that 30 μM SSA decreased 30% cell viability (Supplementary Figure S2), while increased nearly 80% inhibition of the migration. Thus, the antiangiogenic effect of SSA would be from both direct cytotoxicity and the specific interference to the cell function in migration.

HUVEC migration was also investigated using the Transwell test (Luan et al., 2014). The cells were treated with SSA for 10 h. During this time, ∼80% cells were still alive even at the highest test concentration (100 μM) (Supplementary Figure S3). After 10 μM SSA treatment for 10 h, more than 90% HUVECs were alive. Under this treatment condition, more than 60% cell migration was suppressed (Figure 2B). This observation reflected the specific interference of SSA to the cell function in migration. More than 90% migration was suppressed when the cells were treated with 30 and 100 μΜ SSA (Figure 2B).

The tube formation of HUVECs is a classical *in vitro* angiogenesis assay (Luan et al., 2014). The endothelial cells differentiate and form tube-like structures on the extracellular matrix (Matrigel). In contrast to the well-formed tube-like pattern, SSA exhibited strong antiangiogenic potency with more than 90–100% inhibition when concentrations went above 10 μΜ with 8-h treatment (Figure 2C). Specifically, the cells were individually spread on the Matrigel with almost no connection between them.

### SSA Suppressed Angiogenesis in Chick Embryo Chorioallantoic Membrane

**FIGURE 3:**
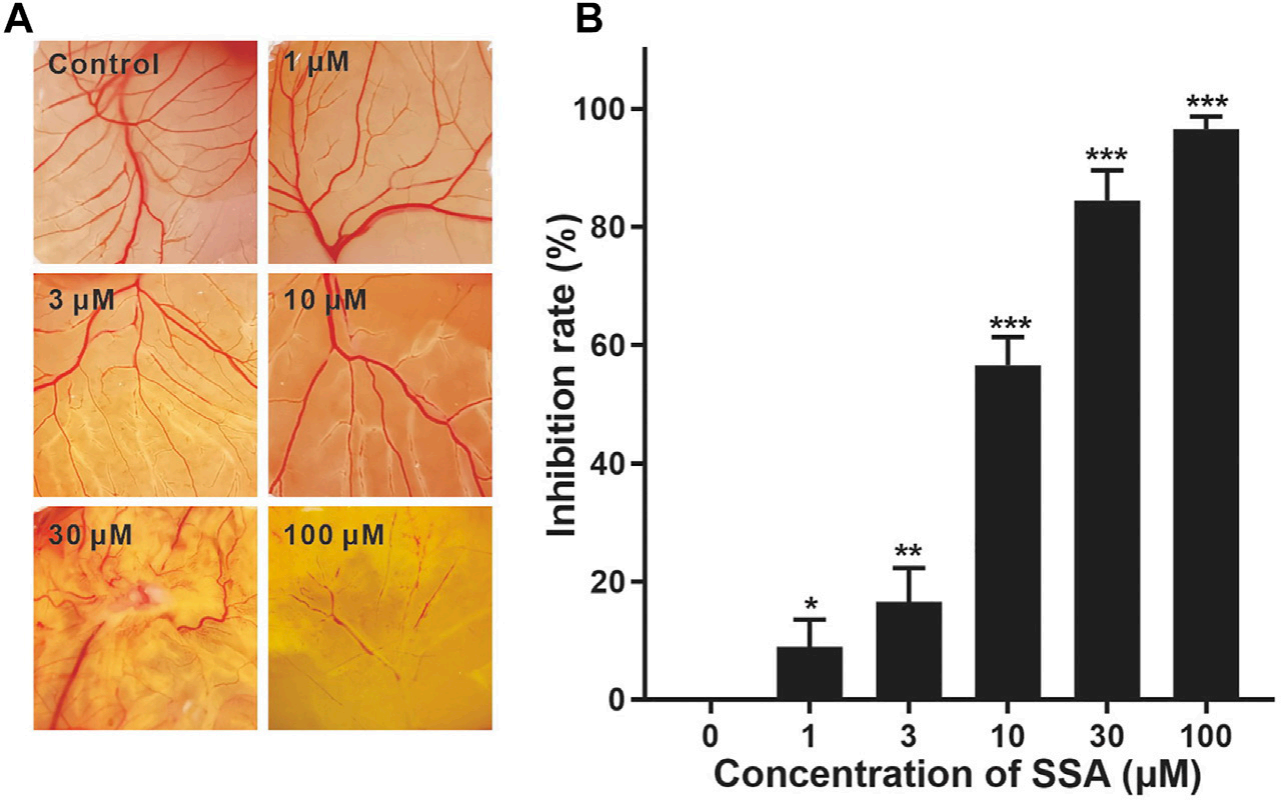
Effect of SSA on angiogenesis in the CAM. CAMs were treated for 48 h with a 6.5-mm diameter Whatman filter disk previously immersed in various concentrations of SSA (1–100 μM). CAM angiogenesis was photographed using a digital camera **(A)** and quantified using Image-Pro Plus 8.0 software **(B)**. Data were expressed as mean ± *s*.d. *n* = 3. *p < 0.05, **p < 0.01, and ***p < 0.001 as compared with the control group. SSA inhibited vascularization in the Matrigel plugs in mice.

We then investigate the antiangiogenic activity of SSA *in vivo* using the classic chick embryo CAM model (Liu et al., 2018). After 48-h treatment, the formation of new blood vessels was dramatically suppressed by SSA compared to that in the control, demonstrating the potent antiangiogenic activity *in vivo* (Figure 3).

### SSA Inhibited Vascularization in the Matrigel Plugs in Mice

**FIGURE 4:**
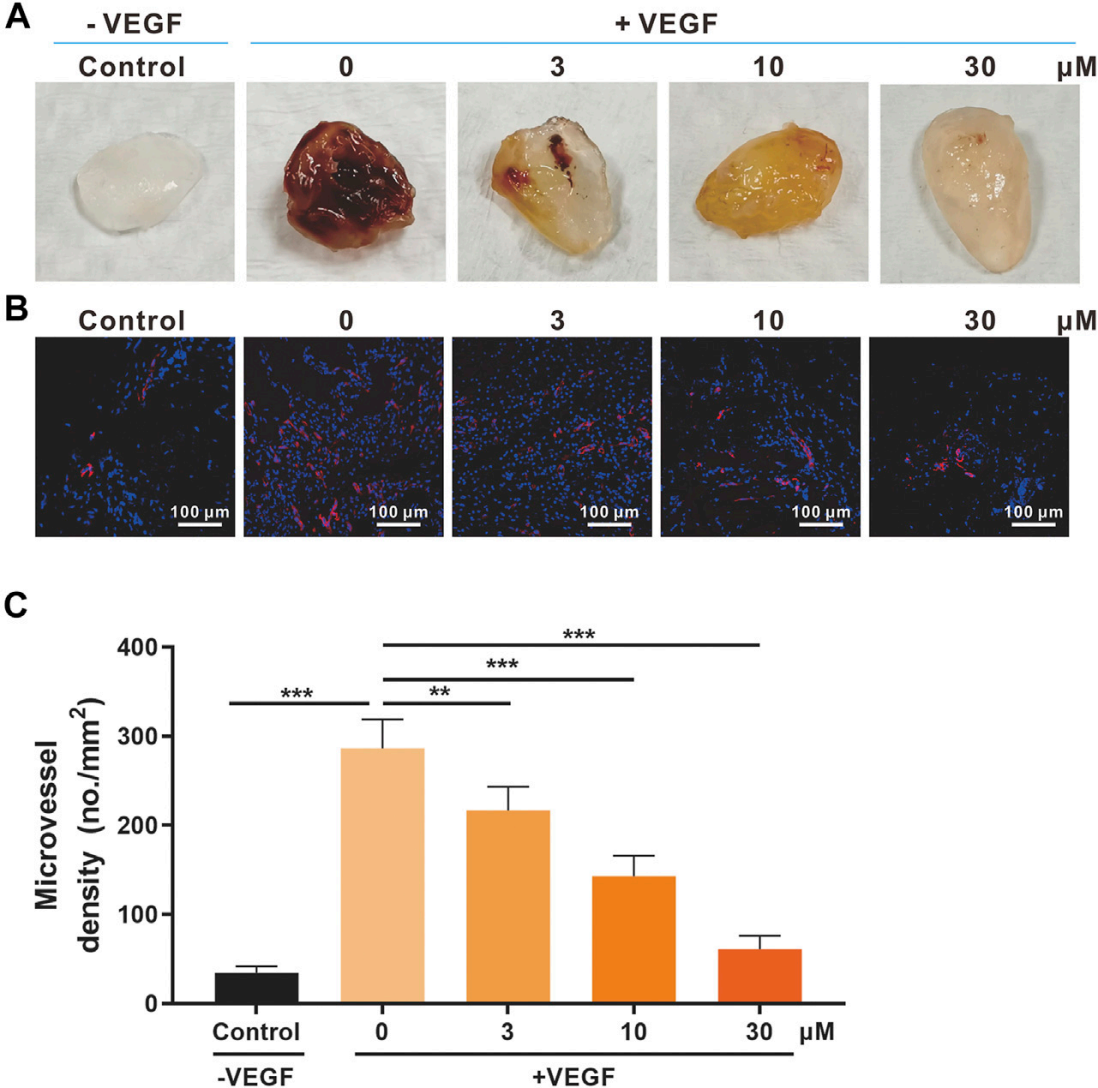
Effects of SSA on *in vivo* angiogenesis evaluated in the Matrigel plug model. **(A)** Photographs of the Matrigel plugs of different groups on day 12. **(B)** Immunofluorescence staining of CD31-positive microvessels (red) in the Matrigel plugs. Representative images were shown. **(C)** Quantified microvessel density (MVD). All values were shown as mean ± *s*.d. *n* = 5. **p < 0.01 and ***p < 0.001. SSA inhibited angiogenesis and growth of orthotopic 4T1 tumors in mice.

The Matrigel plug model was also used for the *in vivo* antiangiogenic evaluation of SSA (Liu et al., 2015). Matrigel containing 30 U heparin, VEGF_165_ (0 or 50 ng/ml), or SSA (3, 10, and 30 μM) was subcutaneously injected into female BALB/c mice. After 12 days, the formed Matrigel plugs were harvested and photographed (Figure 4A). The immunofluorescent staining of CD31-positive blood vessels in the plugs indicated that SSA effectively inhibits angiogenesis in a dose-dependent manner (Figures 4B,C).

### SSA Inhibited Angiogenesis and Growth of Orthotopic 4T1 Tumors in Mice

**FIGURE 5:**
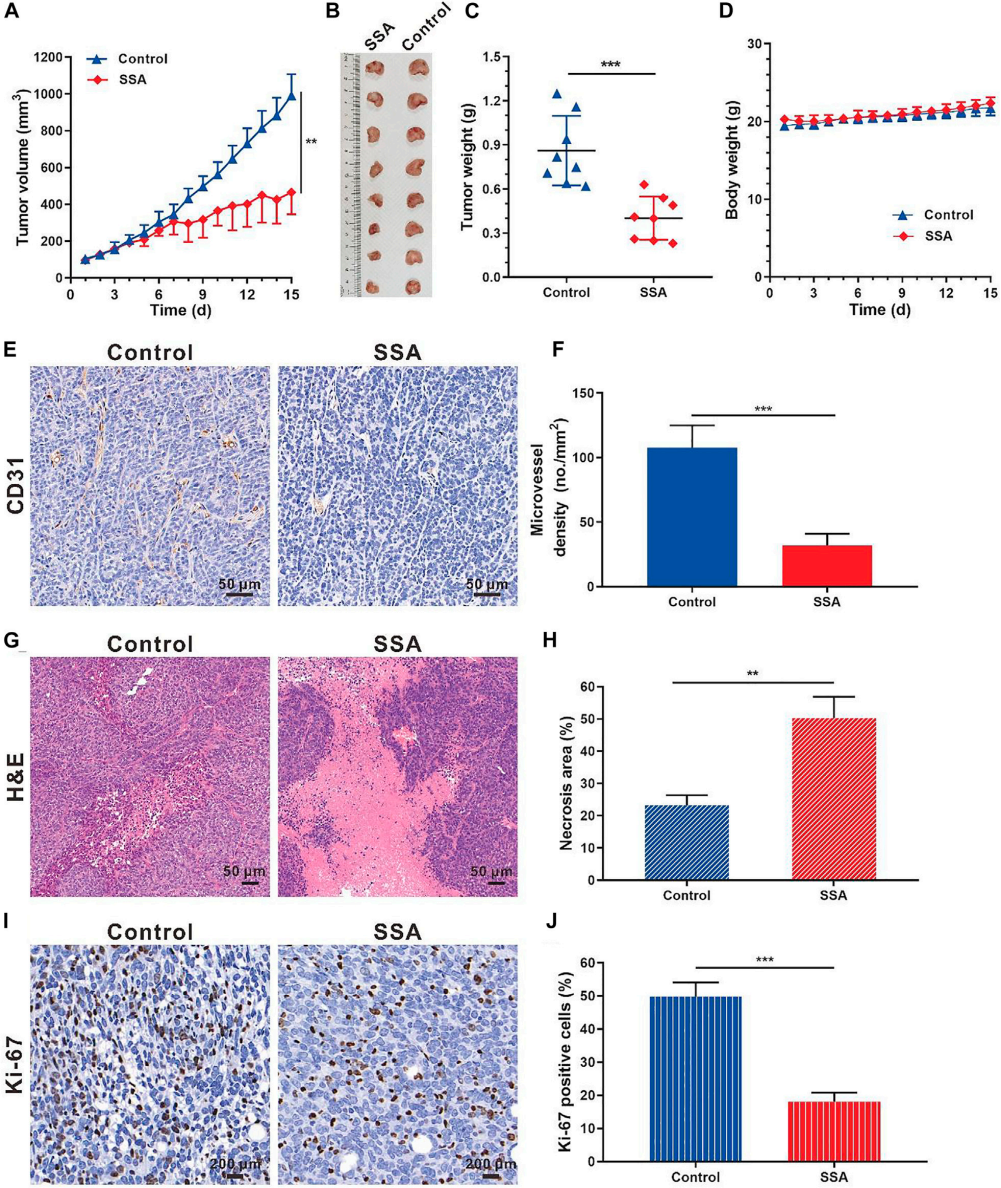
SSA inhibited orthotopic 4T1 tumor growth and angiogenesis in BALB/c mice. When the tumor grew to approximately 100 mm^3^, the mice were treated intraperitoneally with saline or SSA (10 mg/kg/day) for consecutive 15 days. **(A)** Tumor growth curve. **(B)** Photographs of the excised tumor on day 15. **(C)** Tumor weight on day 15. **(D)** Mouse body weight during the therapy. **(E)** Representative images of the immunohistochemical staining of CD31^+^ tumor vessels. **(F)** Quantified analysis of tumor microvessel density (MVD). **(G)** H&E staining of the tumors showed the necrosis region. **(H)** Quantified necrosis area assay. **(I)** The brown nuclei of proliferative tumor cells were stained with anti-Ki-67 antibody. **(J)** Statistical assay of the Ki-67 positive tumor cells in panel I. All values were shown as mean ± *s*.d. *n* = 8 in A, C, and D. *n* = 5 in F, H, and J. **p < 0.01, ***p < 0.001. SSA suppressed angiogenesis and growth of subcutaneous HCT-15 tumors in mice.

Based on the antiangiogenic performance of SSA in chick embryo CAM and Matrigel plug models, we hypothesized that SSA would inhibit tumor growth by suppressing angiogenesis. The orthotopic 4T1 tumor breast tumor model was adopted for this test. Starting from the seventh day, the tumor growth rate of the SSA-treated group slowed down significantly. On day 15 (the end of the test), the tumor volume treated by SSA was 465.1 mm^3^, 53.1% smaller than that (991.4 mm^3^) of the control group (Figures 5A,B). Correspondingly, the tumor weight (0.40 g) of the SSA group was 39.6% less than that (0.86 g) of the control group (Figure 5C). The mouse body weight was well-maintained, indicating good tolerance of the therapeutic regimen (Figure 5D). Immunohistochemical and pathological assays showed that SSA treatment obviously decreased MVD and dramatically elevated the necrosis area than that in the control group (Figures 5E–H). The antitumor effect was also reflected in the percentage of proliferative cells in tumors. It showed that SSA treatment pronounceably reduced the Ki-67–positive tumor cells (Figures 5I,J).

### SSA Suppressed Angiogenesis and Growth of Subcutaneous HCT-15 Tumors in Mice

**FIGURE 6:**
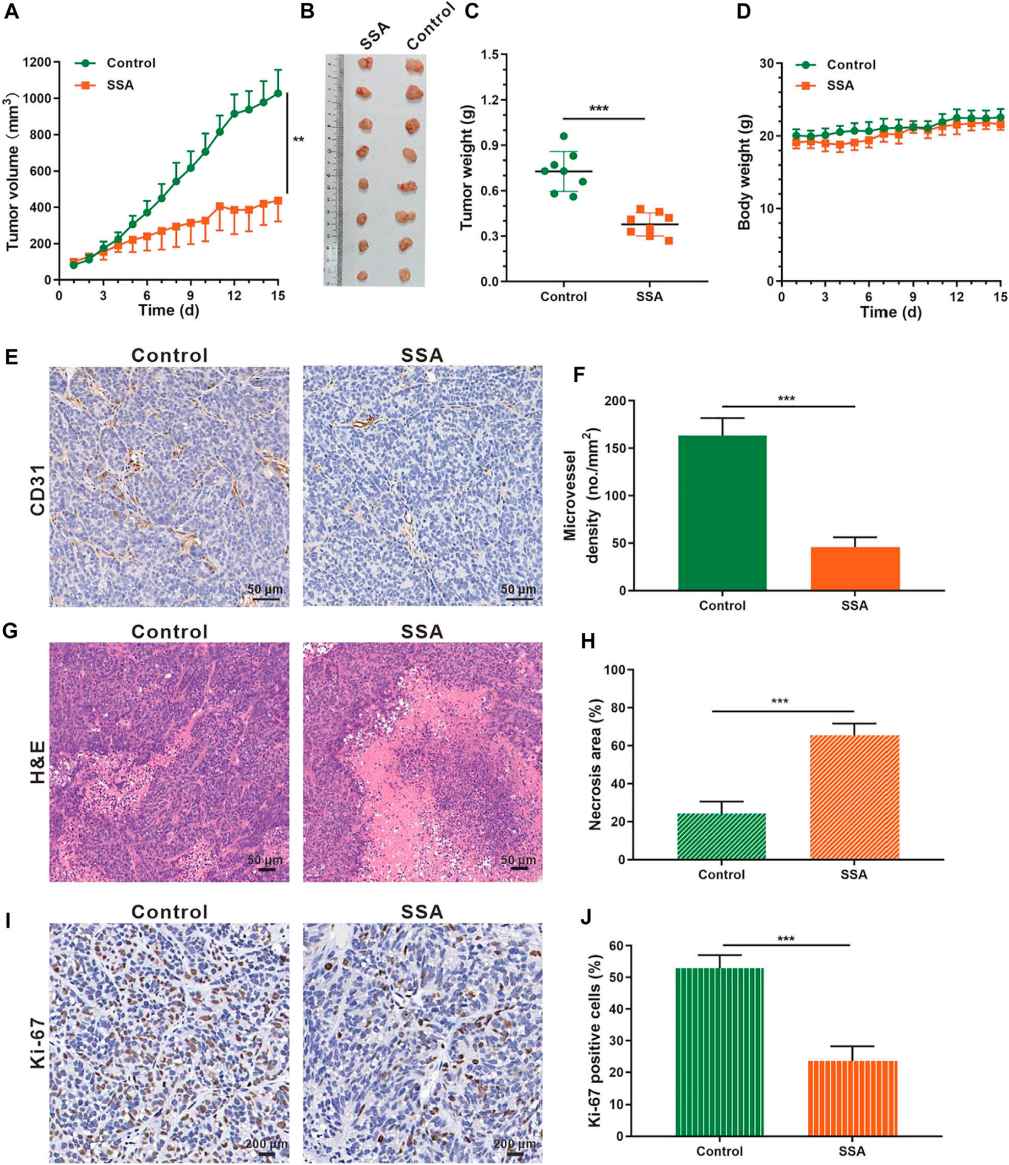
SSA suppressed HCT-15 tumor growth by inhibiting tumor angiogenesis. When the tumor volume reached ∼100 mm^3^, the mice were treated intraperitoneally with saline or SSA (10 mg/kg) every day for consecutive 15 days. **(A)** Tumor growth curve. **(B)** Photographs of the excised tumor on day 15. **(C)** Tumor weight on day 15. **(**D**)** Mouse body weight during the therapy. **(E)** Representative images of the immunohistochemical staining of CD31^+^ tumor vessels. **(F)** Quantified analysis of tumor microvessel density (MVD). **(G)** H&E staining of the tumors showed the necrosis region. **(H)** Quantified necrosis area assay. **(I)** The brown nuclei of proliferative tumor cells were stained with anti–Ki-67 antibody. **(J)** Statistical assay of the Ki-67 positive tumor cells in panel I. All values were shown as mean ± *s*.d. *n* = 8 in A, C, and D. *n* = 5 in F, H, and I. **p < 0.01, ***p < 0.001. SSA inhibited angiogenesis by suppressing the VEGFR2 signaling pathway and Its downstream proteins.

The antiangiogenic activity of SSA was also examined in HCT-15 tumors in mice. Similar antitumor effects like those in 4T1 tumors were obtained. At the end of the test (day 15), the HCT-15 tumor volume treated by SSA was 436.9 mm^3^, 57.5% smaller than that (1,028.2 mm^3^) of the control group (Figures 6A,B). Correspondingly, the tumor weight (0.35 g) of the SSA group was 52.1% less than that (0.73 g) of the control group (Figure 6C). No obvious loss of mouse body weight appeared, demonstrating good safety (Figure 6D). Tumor vessels indicated as MVD were largely inhibited in the SSA-treated group, and the necrosis area of tumor tissues was dramatically increased (Figures 6E–H). Same as the observation as in 4T1 tumors, the proliferative Ki-67–positive cells in HCT-15 tumors were dramatically decreased after SSA treatment, indicating significant antitumor efficacy (Figures 6I,J).

### SSA Inhibited Angiogenesis by Suppressing VEGFR2 Signaling Pathway and Its Downstream Proteins

**FIGURE 7:**
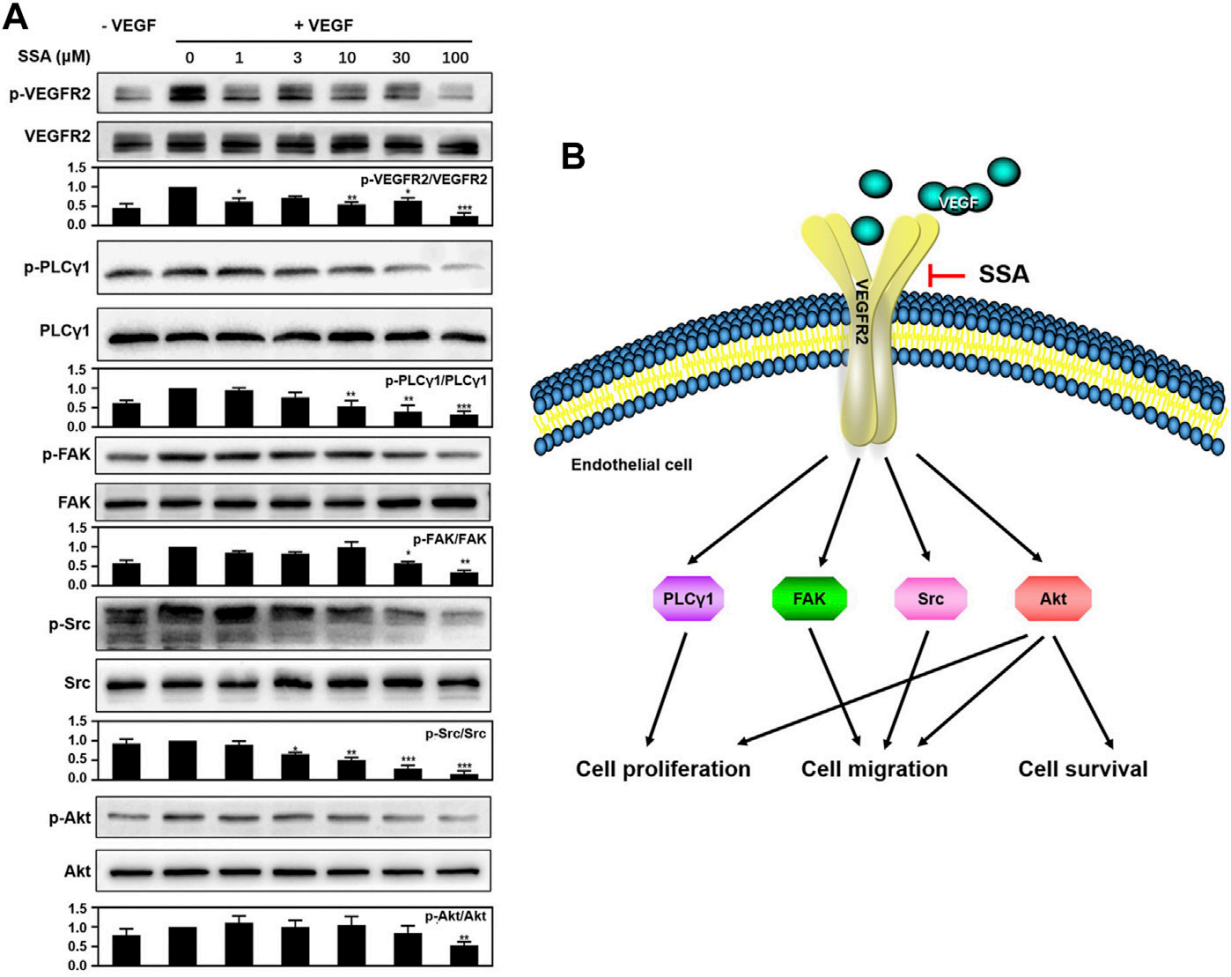
SSA suppressed the phosphorylation of VEGFR2 and downstream signaling molecules in HUVECs. (**A**) SSA inhibited the activation of VEGFR2 and its downstream signaling kinases in HUVECs. The activation of VEGFR2 and its downstream proteins, such as PLCγ1, FAK, Src, and Akt was examined by Western blot. The gray scale ratio of the phosphorylated protein to the total protein was shown. Comparisons to the control group (cell treated only with VEGF_165_) were performed. n = 3. (**B**) Diagram of the SSA-mediated antiangiogenic signaling pathway. *p < 0.05, **p < 0.01, and ***p < 0.001. n = 3.

To explore the mechanism of SSA-mediated antiangiogenic activity in HUVECs, Western blot assay was used to examine whether SSA could inhibit the phosphorylation of VEGFR2 and its downstream proteins, the most important angiogenic signal pathway that regulates the endothelial cell function in angiogenesis. It showed that VEGFR2 activation and the downstream signaling, including PLCγ1, FAK, Src, and Akt, were decreased when treated with different concentrations of SSA in a concentration-dependent manner (Figure 7A).

## Discussion

Antiangiogenic therapy is a major therapeutic modality in cancer clinics. More than 10 antiangiogenic agents have been approved worldwide, and the small molecular kinase inhibitors account for the majority (Jayson et al., 2016). However, the clinical benefits are compromised by the therapy-associated side effects and resistance (Jayson et al., 2016). Moreover, unsatisfied pharmacokinetic behavior and high cost limit their wide use in cancer clinics (Jain et al., 2011; Kim et al., 2019). Exploring novel natural products with antiangiogenic activity is an emerging attractive field (Yang and Wu, 2015; Guan et al., 2016; Kotoku et al., 2016; Lu et al., 2016). Besides similar therapeutic potential, natural products are generally inexpensive and less toxic (Lu et al., 2016).

SSA is a triterpenoid saponin extracted from the traditional Chinese medicinal herb *Radix Bupleurum*. Its diverse pharmacological activities including antitumor effects have been revealed. However, the antiangiogenic potency of SSA still remains unknown. In this work, the antiangiogenic activity of SSA was carefully characterized. Compared to the tumor cells (4T1 and HCT-15), HUVEC viability was more efficiently inhibited by SSA, indicating higher sensitivity of the tumor vascular endothelial cells. SSA also suppressed HUVEC migration and tube formation in a dose-dependent manner. The good antiangiogenic activities are also well-observed in *in vivo* models of the chick embryo CAM and Matrigel plugs. The potent antiangiogenic activity resulted in significant antitumor effects in two solid tumor models, orthotopic 4T1 breast cancer, and subcutaneous HCT-15 colorectal cancer. Over 50% of tumor repression was obtained at dose of 10 mg/kg/day for consecutive 15 injections. Further dose escalation and antitumor effects are warranted.

VEGFR2 signaling is the most important pathway for tumor angiogenesis (Kerbel, 2008). Western blot assay indicated that SSA could decrease the VEGF-induced VEGFR2 phosphorylation and its downstream signaling pathways, including PLCγ1, FAK, Src, and Akt, in a dose-dependent manner. The downregulation of PLCγ1 can be responsible for the inhibition of human vascular endothelial cell (HUVEC) proliferation (Sase et al., 2009). The inactivation of FAK and Src can compromise endothelial cell migration (Guan et al., 2015). Akt involves multiple cellular functions, including cell survival, proliferation, migration, and protein synthesis (Liu et al., 2018). The antiangiogenic molecular mechanism of SSA is summarized in Figure 7B. It is noted that other triterpenoid natural products, such as Raddeanin A (Guan et al., 2015), Platycodin D (Luan et al., 2014), Cucurbitacin E (Dong et al., 2010), and acetyl-11-keto-β-boswellic acid (AKBA) (Pang et al., 2009), also exert antiangiogenic roles via blocking of VEGFR2 signaling. The study of the structure–activity relationship will help reveal the mechanism of action of these compounds in depth.

In conclusion, our findings first revealed that SSA possesses potent antiangiogenic activities, thereby suppressing tumor growth by blocking VEGFR2 signaling pathways. These observations demonstrate that SSA may be a potential drug candidate or lead compound for antiangiogenic cancer therapy.

## Data Availability

The raw data supporting the conclusions of this article will be made available by the authors, without undue reservation.
