# Supplementary material for: Saikosaponin A, a Triterpene Saponin, Suppresses Angiogenesis and Tumor Growth by Blocking VEGFR2-Mediated Signaling Pathway
Source: Front Pharmacol. 2021 Oct 29;12:713200. doi: 10.3389/fphar.2021.713200 (PMC8588445; doi:10.3389/fphar.2021.713200)
Supplement: Supplementary file 1 [file DataSheet1.DOCX]

**Supplementary Materials**

**
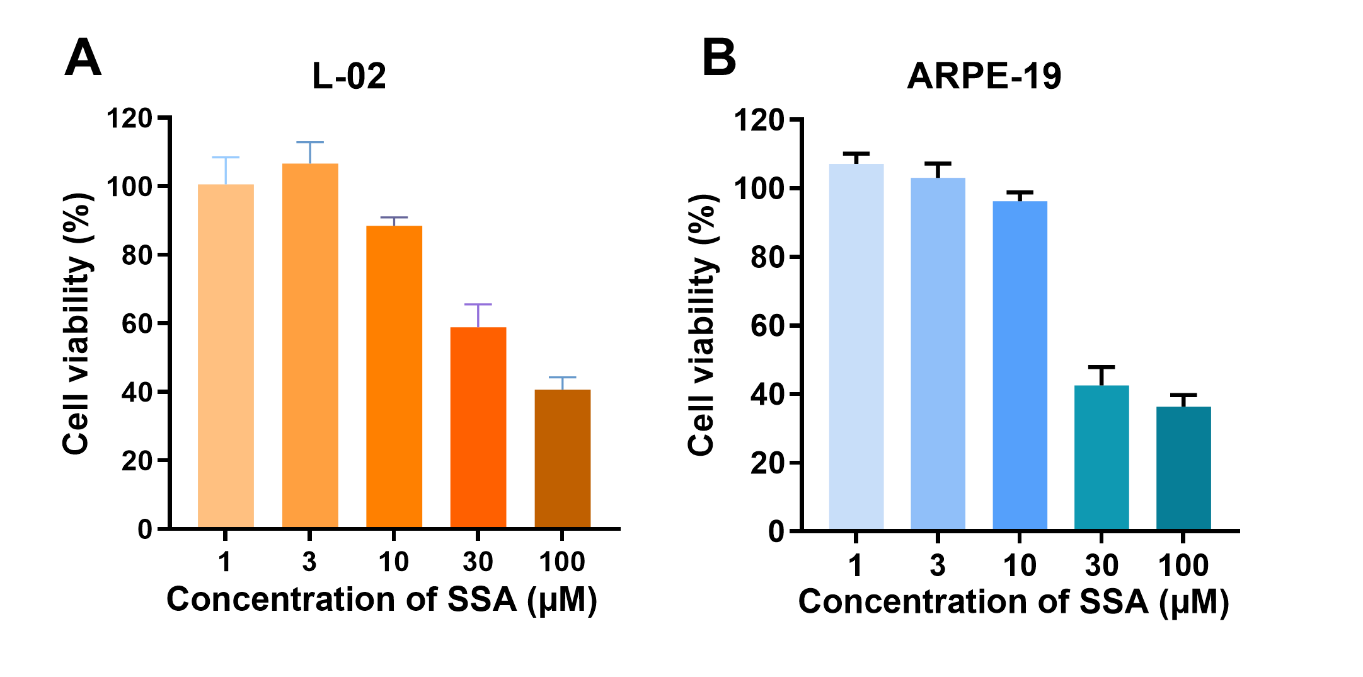
**

**Supplemental Figure 1.** The effect of SSA on normal cell viability. Two normal cell lines, L-02 (human fetal hepatocyte line) and ARPE-19 (a human retinal pigment epithelial cell line), were tested. The cells were incubated with SSA for 48 h, and cell viabilities were examined using CCK-8 assay.


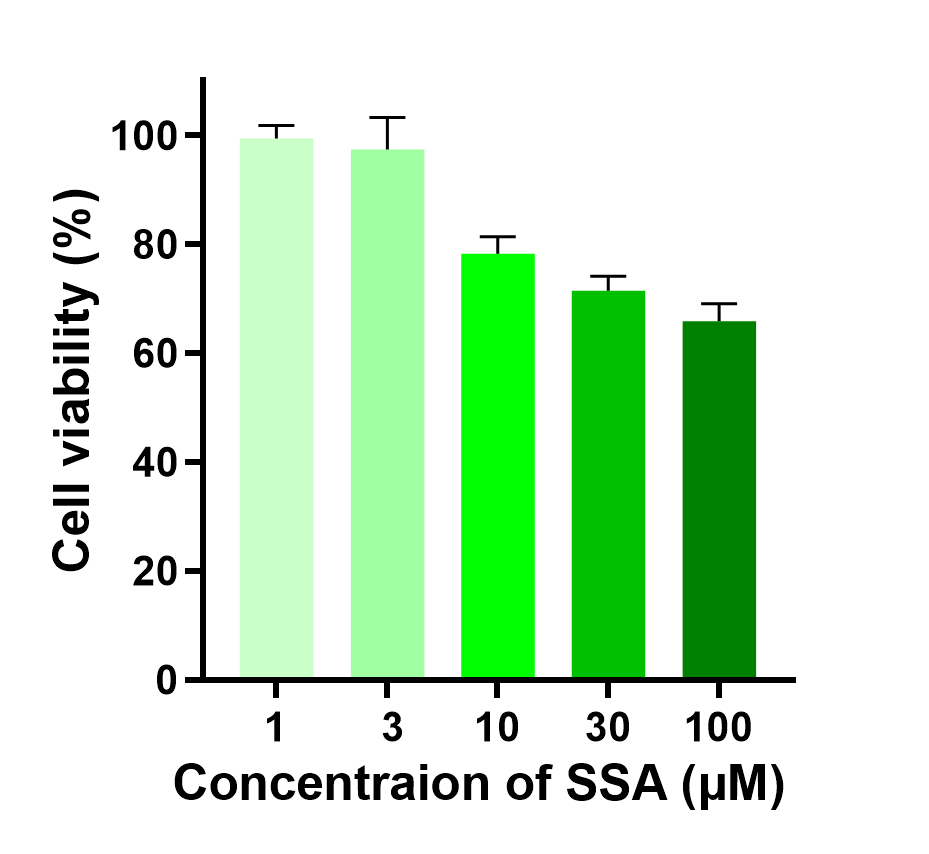


**Supplemental Figure 2.** Cell viability of HUVECs after treatment with SSA for 12 h.


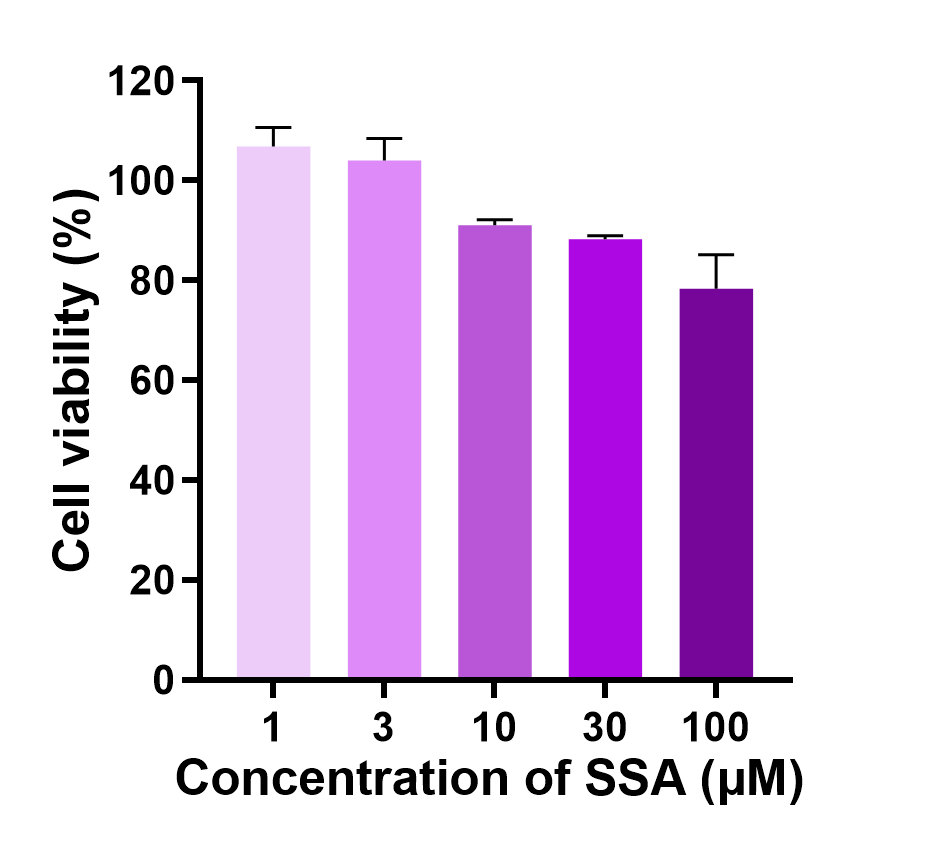


**Supplemental Figure 3.** Cell viability of HUVECs after treatment with SSA for 10 h.
